# A new approach to study the Zeno effect for a macroscopic quantum system under frequent interactions with a harmonic environment

**DOI:** 10.1038/s41598-019-51729-1

**Published:** 2019-10-24

**Authors:** Fatemeh Ghasemi, Afshin Shafiee

**Affiliations:** 1 Research Group on Foundations of Quantum Theory and Information, Department of Chemistry, Sharif University of Technology, P.O.Box 11365-9516, Tehran, Iran; 2 School of Physics, Institute for Research in Fundamental Sciences (IPM), P.O.Box 19395-5531, Tehran, Iran

**Keywords:** Theoretical physics, Quantum mechanics

## Abstract

Quantum Zeno and anti-Zeno behaviors of a two-level macroscopic quantum system in interaction with a harmonic environment are studied using the perturbation theory. The system-environment interactions are applied in a successive and step-by-step way. A new expression for the probability of surviving the macrosystem in its initial state, after the *N*th interaction step, is derived through a different method of calculation. It is shown that multiple transitions between Zeno and anti-Zeno behaviors can be found in our approach. We have shown that in addition to the environmental parameters like the Ohmicity, the macroscopic trait of the system also has a notable effect on the decay rate Γ(*τ*). Moreover, we have investigated how the decay rate Γ(*τ*) varies as the parameter of the macroscopicity of the system $\tilde{{\boldsymbol{h}}}$ changes continuously in a given domain.

## Introduction

The quantum Zeno effect (QZE) is stated to be a phenomenon in which the time evolution of a given quantum state slows down due to the frequent measurements of the system^1–14^. This phenomenon seems to be opposed to the well-known measurement problem. Misra and Sudarshan were the first researchers who found this paradoxical behavior through their theoretical investigations in 1977^1^. On the other hand, the frequent measurements may have a reverse effect and accelerate the evolution under somewhat different conditions. This opposite effect is called the quantum anti-Zeno effect (QAZE) and occurs when the frequent measurements are not rapid enough^3,15–20^.

The QZE and QAZE are considered to be interesting general features of quantum mechanics. Up to now, both QZE and QAZE have attracted attention from both theoretical and experimental point of views. Since the discovery, the QZE and QAZE, have been studied in many different physical contexts such as radiative decay^21^, dynamical control of tunneling^18,22^, Bose-Einstein condensates^23^, protecting quantum information^10^, quantum transport in disordered systems^19^, and thermodynamical control and state-purification of quantum systems^24^.

Fundamentally, these effects are important at least from two perspectives: they could be critical to study the dynamics of open quantum systems^25–28^ and are also expected to shed light on the well-known measurement problem which is still an open problem^29–32^. Presilla and co-workers in 1996, through a generic work, put together all the five developed approaches to the measurement problem and showed that they are mathematically equivalent and lie in two selective and nonselective measurement classifications. Alternatively, through an example of QZE, they could introduce the measurement couplings as phenomenological parameters inferred in comparison with experimental data^33^.

These effects were under-study in the framework of microscopic systems, till Barone and co-workers succeeded to control the Zeno and anti-Zeno behavior of a macroscopic quantum system in 2004^18^. In 2010, Paolo Facchi and co-workers studied the classical limit of the QZE in a simple formalism, namely the position measurements applied to a free particle. By a semiclassical analysis they concluded that the QZE is a purely quantum phenomenon without classical analog^34^. Though for a long time, the investigations of QZE and QAZE had mainly focused on the population decay of a quantum system coupled to an environment^3,16,20,35–37^, Chaudhry and co-worker switched to also examine the effect of dephasing in their investigation in 2014^38,39^.

In this article, we return to Barone’s view and study what happens to a macroscopic quantum system under the successive and step-by-step interactions with a harmonic environment. In section II, we introduce a new approach to investigate Zeno and anti-Zeno behaviors throughout a distinct calculation method. We suppose that the system-environment interaction is weak enough so that the perturbation theory applies. In section III, we derive an expression for the probability of finding the macrosystem in the initial state, after the *N*th interaction step. In section IV we show how the macroscopic trait of the system affects the behavior of the decay rate Γ(*τ*). Finally, in section V, we briefly conclude our obtained results.

## Calculations

We discuss the consecutive impacts that a harmonic oscillating environment has on a two-level macroscopic system, to study the Zeno dynamics of the system. We start by representing the system-environment Hamiltonian as

1 $$\hat{H}={\hat{H}}_{s}+{\hat{H}}_{\varepsilon }+{\hat{H}}_{s\varepsilon }$$

where *H*_*s*_ and *H*_*ε*_ are the system and the environment Hamiltonians, respectively. *H*_*sε*_ is the system-environment interaction Hamiltonian which in our formalism^40,41^ has the form

2 $${\hat{H}}_{s\varepsilon }=-\sqrt{\frac{\tilde{h}}{2}}\sum _{\alpha }\,{\omega }_{\alpha }^{3/2}{f}_{\alpha }(\hat{q})({\hat{b}}_{\alpha }+{\hat{b}}_{\alpha }^{\dagger })+\frac{1}{2}\sum _{\alpha }\,{\omega }_{\alpha }^{2}{\{{f}_{\alpha }(\hat{q})\}}^{2}$$

where *q* represents the position variable of system, *ω*_*α*_ is the frequency of the harmonic oscillator of the environment, ${\hat{b}}_{\alpha }^{\dagger }$ and ${\hat{b}}_{a}$ are the creation and annihilation operators for the oscillators and ${f}_{a}(\hat{q})$ describes how the particle *q* couples to the *α*th environment mode. Here, we use a linearly coupled harmonic environment model, named’separable model’ in which ${f}_{a}(\hat{q})={\gamma }_{a}f(\hat{q})$, where $f(\hat{q})$ is an arbitrary function of *q* and *γ*_*α*_ is a positive constant. In Eq. (2) all variables are dimensionless, note that $\tilde{h}$ is also the dimensionless Planck constant which will be introduced in details in section IV.

Suppose that the initial state of the two-level macrosystem is $|\psi (0)\rangle =\frac{1}{\sqrt{2}}(|0\rangle +|1\rangle )$ and that of the environment is |vac〉. Accordingly, the initial state of the entire system is

3 $$|\Psi (0)\rangle \rangle =|\psi ,{\rm{vac}}\rangle \rangle =|\psi \rangle |{\rm{vac}}\rangle =\frac{1}{\sqrt{2}}(|0\rangle +|1\rangle )|{\rm{vac}}\rangle $$

**Figure 1:**
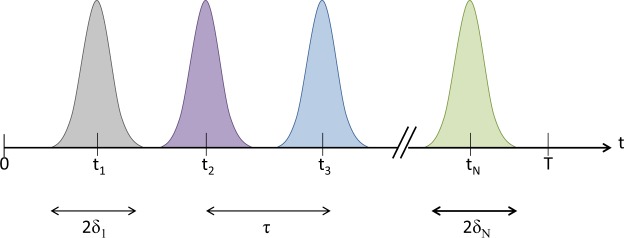
Discrete interactions between the macrosystem and the environment.

We suppose that the macrosystem-environment interactions are time-discrete and happen step by step, known as repeated measurements. Moreover, the time steps are separated by a constant interval of *τ* and the impact width of the interaction *δ* is also narrow enough, i.e.

4 $${t}_{j}=j\tau ,{\delta }_{j}=\delta \ll {\tau }_{s}$$

where *j* specifies the number of steps, *δ* is the half-width of the interaction peak according to Fig. (1), and *τ*_*s*_ is the characteristic timescale associated with the intrinsic time evolution of a macrosystem in absence of the environment. For a two-level macrosystem with the basis states {|0〉, |1〉} we have *τ*_*s*_ = Δ_*s*_^−1^, where Δ_*s*_ depends on the energy difference between the states.

Now let us track the evolution of the entire system step by step. According to the Fig. 1, since $\hat{H}={\hat{H}}_{s}$ in the time interval 0 < *t* < *τ* − *δ*, the evolution of the entire system follows from $|\Psi (t)\rangle \rangle =\hat{U}(t)|\Psi (0)\rangle \rangle$, where $\hat{U}(t)$ is the unitary time evolution operator. The system state at time *τ* − *δ* is then

5 $$|\Psi (\tau -\delta )\rangle \rangle ={e}^{-{\rm{i}}(\tau -\delta ){\hat{H}}_{s}/h}|\psi (0)\rangle |{\rm{vac}}\rangle =\frac{1}{\sqrt{2}}{e}^{-{\rm{i}}\tau {E}_{0}/h}|0\rangle |{\rm{vac}}\rangle +\frac{1}{\sqrt{2}}{e}^{-{\rm{i}}\tau {E}_{1}/h}|1\rangle |{\rm{vac}}\rangle $$

where the coefficients *e*^−i*δE*^_0_^/*h*^ and *e*^−i*δE*^_1_^/*h*^ are approximated by 1, according to the condition stated in Eq. (4).

Regarding once again the Fig. 1, we see that in the time interval *τ* − *δ* < *t* < *τ* + *δ* the macrosystem-environment interaction holds, as described by Eq. 2. In order to investigate the time evolution of the entire system with the initial state |Ψ(*τ* − *δ*)〉〉, we apply the time evolution operator in the interaction picture ${\hat{U}}_{I}(\tau +\delta )={e}^{-i{\hat{H}}_{s\varepsilon }t/\tilde{h}}$, so that we may write

6 $$|\Psi (\tau +\delta )\rangle \rangle ={\hat{U}}_{I}(\tau +\delta )|\Psi (\tau -\delta )\rangle \rangle $$

Using the completeness relation $\sum |n\rangle \langle n|=1$, where {|*n*〉|*n* = 0, 1} are the basis states of the Hilbert space of the system ${ {\mathcal H} }_{s}s$, we rewrite the Eq. (6) as

7 $$|\Psi (\tau +\delta )\rangle \rangle =\sum _{n}|n\rangle \langle n|{\hat{U}}_{I}(\tau +\delta )|\Psi (\tau -\delta )\rangle \rangle =\sum _{n}|n\rangle |{\chi }_{n}(\tilde{\tau }+\delta )\rangle $$

where the states $|{\chi }_{n}(\tilde{\tau }+\delta )\rangle$ are time-dependent coefficients belonging to the Hilbert space of the environment ${ {\mathcal H} }_{\varepsilon }$, with the following definition

8 $$|{\chi }_{n}(\tilde{\tau }+\delta )\rangle =\langle n|{\hat{U}}_{I}(\tau +\delta )|\Psi (\tau -\delta )\rangle \rangle $$

In order to calculate the coefficients $|{\chi }_{n}(\tilde{\tau }+\delta )\rangle$, we resort to the perturbation theory, which can be used when the system-environment interaction is weak. Accordingly, we can expand the time-evolution operator, regarding the interaction Hamiltonian ${\tilde{H}}_{s\varepsilon }$ up to the second order to find

9 $$\begin{array}{rcl}{\hat{U}}_{I}(t) & \simeq  & 1-\frac{i}{\tilde{h}}{\int }_{0}^{t}\,{\rm{d}}{t}_{1}{\hat{H}}_{s\varepsilon }({t}_{1})\\  &  & -\frac{1}{{\tilde{h}}^{2}}{\int }_{0}^{t}\,{\rm{d}}{t}_{2}{\int }_{0}^{{t}_{2}}\,{\rm{d}}{t}_{1}{\hat{H}}_{s\varepsilon }({t}_{2}){\hat{H}}_{s\varepsilon }({t}_{1})\end{array}$$

where the second and third terms of the right-hand side in Eq. (9) are the first and second order correlations, respectively. Let us now go back to the assumption |Ψ(0)〉 = |*ψ*〉|vac〉 and evaluate the expressions ${\tilde{H}}_{s\varepsilon }({t}_{1})|{\rm{vac}}\rangle$ and ${\tilde{H}}_{s\varepsilon }({t}_{2}){\tilde{H}}_{s\varepsilon }({t}_{1})|{\rm{vac}}\rangle$ to specify the coefficients $|{\chi }_{n}(\tilde{\tau }+\delta )\rangle$. Doing so, we arrive at

10 $${\hat{U}}_{I}(\tau +\delta )|{\rm{vac}}\rangle \simeq {\hat{u}}_{{\rm{vac}}}(\tau +\delta )|{\rm{vac}}\rangle +\sum _{\alpha }{\hat{u}}_{\alpha }(\tau +\delta )|\alpha \rangle $$

The detailed forms of the operators ${\hat{u}}_{{\rm{vac}}}$ and ${\hat{u}}_{a}$ are given in Appendix A. Finally using Eq. (10) one can evaluate the coefficients $|{\chi }_{n}(\tilde{\tau }+\delta )\rangle$ in Eq. (8) as

11 $$|{\chi }_{n}(\tilde{\tau }+\delta )\rangle =|{\rm{vac}}\rangle \langle n|{\hat{u}}_{{\rm{vac}}}(\tau +\delta )|\psi \rangle +\sum _{\alpha }|\alpha \rangle \langle n|{\hat{u}}_{\alpha }(\tau +\delta )|\psi \rangle $$

Recall that for the two-level system, *n* takes the values 0 or 1. So the coefficients $|{\chi }_{0}(\tilde{\tau }+\delta )\rangle$ and $|{\chi }_{1}(\tilde{\tau }+\delta )\rangle$ using Eq. (11) take the following forms, respectively

12a $$|{\chi }_{0}(\tilde{\tau }+\delta )\rangle =\frac{1}{\sqrt{2}}{e}^{-{\rm{i}}\tau {E}_{0}/h}\langle 0|{\hat{u}}_{{\rm{vac}}}(\tau +\delta )|0\rangle |{\rm{vac}}\rangle -\frac{1}{\sqrt{2}}{e}^{-{\rm{i}}\tau {E}_{1}/h}\sum _{\alpha }\langle 0|{\hat{u}}_{\alpha }(\tau +\delta )|1\rangle |\alpha \rangle $$

12b $$|{\chi }_{1}(\tilde{\tau }+\delta )\rangle =\frac{1}{\sqrt{2}}{e}^{-{\rm{i}}\tau {E}_{0}/h}\sum _{\alpha }\,\langle 1|{\hat{u}}_{\alpha }(\tau +\delta )|0\rangle |\alpha \rangle -\frac{1}{\sqrt{2}}{e}^{-{\rm{i}}\tau {E}_{1}/h}\langle 1|{\hat{u}}_{{\rm{vac}}}(\tau +\delta )|1\rangle |{\rm{vac}}\rangle $$

Finally, at the end of this step, putting the last two Eqs (12a) and (12b) together in Eq. (7), the entire system state at time (*τ* + *δ*) can be written as

13 $$\begin{array}{rcl}|\Psi (\tau +\delta )\rangle \rangle  & = & \frac{1}{\sqrt{2}}{e}^{-{\rm{i}}\tau {E}_{0}/h}\langle 0|{\hat{u}}_{{\rm{vac}}}(\tau +\delta )|0\rangle |0\rangle |{\rm{vac}}\rangle \\  &  & -\frac{1}{\sqrt{2}}{e}^{-{\rm{i}}\tau {E}_{1}/h}\sum _{\alpha }\,\langle 0|{\hat{u}}_{\alpha }(\tau +\delta )|1\rangle |0\rangle |\alpha \rangle \\  &  & +\frac{1}{\sqrt{2}}{e}^{-{\rm{i}}\tau {E}_{0}/h}\sum _{\alpha }\,\langle 1|{\hat{u}}_{\alpha }(\tau +\delta )|0\rangle |1\rangle |\alpha \rangle \\  &  & -\frac{1}{\sqrt{2}}{e}^{-{\rm{i}}\tau {E}_{1}/h}\langle 1|{\hat{u}}_{{\rm{vac}}}(\tau +\delta )|1\rangle |1\rangle |{\rm{vac}}\rangle \end{array}$$

Now during the third step, i.e., within the time interval *τ* + *δ* < *t* < 2*τ* − *δ*, we quiet the interaction Hamiltonian as shown in Fig. 1. Once again just like the first step, the effective Hamiltonian is the operator ${\hat{H}}_{s}$. Hence, we find that |Ψ(2*τ* − *δ*)〉〉 is

14 $$\begin{array}{rcl}|\Psi (2\tau -\delta )\rangle \rangle  & = & {e}^{-{\rm{i}}\tau {\hat{H}}_{s}/h}|\Psi (\tau +\delta )\rangle \rangle \\  & = & +\frac{1}{\sqrt{2}}{e}^{-{\rm{i}}\tau {E}_{0}/h}{e}^{-{\rm{i}}\tau {E}_{0}/h}\langle 0|{\hat{u}}_{{\rm{vac}}}(\tau +\delta )|0\rangle |0\rangle |{\rm{vac}}\rangle \\  &  & -\frac{1}{\sqrt{2}}{e}^{-{\rm{i}}\tau {E}_{0}/h}{e}^{-{\rm{i}}\tau {E}_{1}/h}\sum _{\alpha }\,\langle 0|{\hat{u}}_{\alpha }(\tau +\delta )|1\rangle |0\rangle |\alpha \rangle \\  &  & +\frac{1}{\sqrt{2}}{e}^{-{\rm{i}}\tau {E}_{0}/h}{e}^{-{\rm{i}}\tau {E}_{1}/h}\sum _{\alpha }\,\langle 1|{\hat{u}}_{\alpha }(\tau +\delta )|0\rangle |1\rangle |\alpha \rangle \\  &  & -\frac{1}{\sqrt{2}}{e}^{-{\rm{i}}\tau {E}_{1}/h}{e}^{-{\rm{i}}\tau {E}_{1}/h}\langle 1|{\hat{u}}_{{\rm{vac}}}(\tau +\delta )|1\rangle |1\rangle |{\rm{vac}}\rangle \end{array}$$

During the next step in the time interval 2*τ* − *δ* < *t* < 2*τ δ*, we settle the system-environment interaction once again as shown in Fig. 1. To calculate the state |Ψ(2*τ* + *δ*)〉〉, we first identify the essential expressions for $|{\chi }_{0}(\tilde{2\tau }+\delta )\rangle$ and $|{\chi }_{0}(\tilde{2\tau }+\delta )\rangle$ using the Eq. (8) as

15 $$\begin{array}{rcl}|{\chi }_{0}(\tilde{2\tau }+\delta )\rangle  & = & \langle 0|{\hat{U}}_{I}(2\tau +\delta )|\Psi (2\tau -\delta )\rangle \rangle \\  & = & +\frac{1}{\sqrt{2}}{e}^{-{\rm{i}}\tau {E}_{0}/h}{e}^{-{\rm{i}}\tau {E}_{0}/h}\langle 0|{\hat{u}}_{{\rm{vac}}}(\tau +\delta )|0\rangle \langle 0|{\hat{U}}_{I}|0\rangle |{\rm{vac}}\rangle \\  &  & -\frac{1}{\sqrt{2}}{e}^{-{\rm{i}}\tau {E}_{0}/h}{e}^{-{\rm{i}}\tau {E}_{1}/h}\sum _{\alpha }\,\langle 0|{\hat{u}}_{\alpha }(\tau +\delta )|1\rangle \langle 0|{\hat{U}}_{I}|0\rangle |\alpha \rangle \\  &  & +\frac{1}{\sqrt{2}}{e}^{-{\rm{i}}\tau {E}_{0}/h}{e}^{-{\rm{i}}\tau {E}_{1}/h}\sum _{\alpha }\,\langle 1|{\hat{u}}_{\alpha }(\tau +\delta )|0\rangle \langle 0|{\hat{U}}_{I}|1\rangle |\alpha \rangle \\  &  & -\frac{1}{\sqrt{2}}{e}^{-{\rm{i}}\tau {E}_{1}/h}{e}^{-{\rm{i}}\tau {E}_{1}/h}\langle 1|{\hat{u}}_{{\rm{vac}}}(\tau +\delta )|1\rangle \langle 0|{\hat{U}}_{I}|1\rangle |{\rm{vac}}\rangle \end{array}$$

and

16 $$\begin{array}{rcl}|{\chi }_{1}(\tilde{2\tau }+\delta )\rangle  & = & \langle 0|{\hat{U}}_{I}(2\tau +\delta )|\Psi (2\tau -\delta )\rangle \rangle \\  & = & +\frac{1}{\sqrt{2}}{e}^{-{\rm{i}}\tau {E}_{0}/h}{e}^{-{\rm{i}}\tau {E}_{0}/h}\langle 0|{\hat{u}}_{{\rm{vac}}}(\tau +\delta )|0\rangle \langle 1|{\hat{U}}_{I}|0\rangle |{\rm{vac}}\rangle \\  &  & -\frac{1}{\sqrt{2}}{e}^{-{\rm{i}}\tau {E}_{0}/h}{e}^{-{\rm{i}}\tau {E}_{1}/h}\sum _{\alpha }\,\langle 0|{\hat{u}}_{\alpha }(\tau +\delta )|1\rangle \langle 1|{\hat{U}}_{I}|0\rangle |\alpha \rangle \\  &  & +\frac{1}{\sqrt{2}}{e}^{-{\rm{i}}\tau {E}_{0}/h}{e}^{-{\rm{i}}\tau {E}_{1}/h}\sum _{\alpha }\,\langle 1|{\hat{u}}_{\alpha }(\tau +\delta )|0\rangle \langle 1|{\hat{U}}_{I}|1\rangle |\alpha \rangle \\  &  & -\frac{1}{\sqrt{2}}{e}^{-{\rm{i}}\tau {E}_{1}/h}{e}^{-{\rm{i}}\tau {E}_{1}/h}\langle 1|{\hat{u}}_{{\rm{vac}}}(\tau +\delta )|1\rangle \langle 1|{\hat{U}}_{I}|1\rangle |{\rm{vac}}\rangle \end{array}$$

here, to evaluate the states $|{\chi }_{0}(\tilde{2\tau }+\delta )\rangle$ and $|{\chi }_{1}(\tilde{2\tau }+\delta )\rangle$, we face with the expressions ${\hat{U}}_{I}$(2*τ* + *δ*)|vac〉 and ${\hat{U}}_{I}$(2*τ* + *δ*)|*α*〉. We determine the former using Eq. (10). For the latter, we seek the result of applying the operator ${\hat{U}}_{I}$*(*2*τ* + *δ*) to the environmental states |*α*〉. Using Eq. (9), one gets

17 $${\hat{U}}_{I}(2\tau +\delta )|\alpha \rangle \simeq {\hat{u}^{\prime} }_{{\rm{vac}}}(2\tau +\delta )|{\rm{vac}}\rangle +\sum _{\alpha }\,{\hat{u}^{\prime} }_{\alpha }(2\tau +\delta )|\alpha \rangle $$

where the forms of the operators ${\hat{u}^{\prime} }_{vac}$ and ${\hat{u}^{\prime} }_{a}$ are given in Appendix A. According to the Eqs (7), (15), (16) and (17), now we can calculate the system state at time (2*τ* + *δ*) as

18 $$\begin{array}{rcl}|\Psi (2\tau +\delta )\rangle \rangle  & = & +\frac{1}{\sqrt{2}}{e}^{-{\rm{i}}\tau {E}_{0}/h}{e}^{-{\rm{i}}\tau {E}_{0}/h}\langle 0|{\hat{u}}_{{\rm{vac}}}(\tau +\delta )|0\rangle \langle 0|{\hat{u}}_{{\rm{vac}}}(\tau +\delta )|0\rangle |0\rangle |{\rm{vac}}\rangle \\  &  & -\frac{1}{\sqrt{2}}{e}^{-{\rm{i}}\tau {E}_{0}/h}{e}^{-{\rm{i}}\tau {E}_{1}/h}\sum _{\alpha }\,\langle 0|{\hat{u}}_{\alpha }(\tau +\delta )|1\rangle \langle 0|{\hat{u}^{\prime} }_{\alpha }(2\tau +\delta )|0\rangle |0\rangle |\alpha \rangle \\  &  & +\frac{1}{\sqrt{2}}{e}^{-{\rm{i}}\tau {E}_{0}/h}{e}^{-{\rm{i}}\tau {E}_{1}/h}\langle 1|{\hat{u}}_{\alpha }(\tau +\delta )|0\rangle \langle 0|{\hat{u}^{\prime} }_{{\rm{vac}}}(2\tau +\delta )|1\rangle |0\rangle |{\rm{vac}}\rangle \\  &  & -\frac{1}{\sqrt{2}}{e}^{-{\rm{i}}\tau {E}_{1}/h}{e}^{-{\rm{i}}\tau {E}_{1}/h}\langle 1|{\hat{u}}_{{\rm{vac}}}(\tau +\delta )|1\rangle \langle 0|{\hat{u}}_{\alpha }(2\tau +\delta )|1\rangle |0\rangle |{\rm{vac}}\rangle \\  &  & +\frac{1}{\sqrt{2}}{e}^{-{\rm{i}}\tau {E}_{0}/h}{e}^{-{\rm{i}}\tau {E}_{0}/h}\sum _{\alpha }\,\langle 0|{\hat{u}}_{{\rm{vac}}}(\tau +\delta )|0\rangle \langle 1|{\hat{u}}_{\alpha }(2\tau +\delta )|0\rangle |1\rangle |\alpha \rangle \\  &  & -\frac{1}{\sqrt{2}}{e}^{-{\rm{i}}\tau {E}_{0}/h}{e}^{-{\rm{i}}\tau {E}_{1}/h}\langle 0|{\hat{u}}_{\alpha }(\tau +\delta )|1\rangle \langle 1|{\hat{u}^{\prime} }_{{\rm{vac}}}(2\tau +\delta )|0\rangle |1\rangle |{\rm{vac}}\rangle \\  &  & +\frac{1}{\sqrt{2}}{e}^{-{\rm{i}}\tau {E}_{0}/h}{e}^{-{\rm{i}}\tau {E}_{1}/h}\sum _{\alpha }\,\langle 1|{\hat{u}}_{\alpha }(\tau +\delta )|0\rangle \langle 1|{\hat{u}^{\prime} }_{\alpha }(2\tau +\delta )|1\rangle |1\rangle |\alpha \rangle \\  &  & -\frac{1}{\sqrt{2}}{e}^{-{\rm{i}}\tau {E}_{1}/h}{e}^{-{\rm{i}}\tau {E}_{1}/h}\langle 1|{\hat{u}}_{{\rm{vac}}}(\tau +\delta )|1\rangle \langle 1|{\hat{u}}_{{\rm{vac}}}(\tau +\delta )|1\rangle |1\rangle |{\rm{vac}}\rangle \end{array}$$

## The ***N***th Interaction Step

After the implementation of successive steps, now let us investigate if the macrosystem could show the Zeno behavior. Actually after each interaction step, we can examine the probability of getting the same result as the initial state. However, the ultimate purpose is to derive a relation, which confirms that the macrosystem is in the initial state after all *N* interaction steps, i.e., after the time *t* = *Nτ*. Here, we first derive the relations for the probability of finding the macrosystem in the initial state at times *τ* + *δ*, 2*τ* + *δ*, 3*τ* + *δ* and 4*τ* + *δ*. For instance, the probability amplitude at time 2*τ* + *δ* (we have chosen *t* = 2*τ* + *δ* due to the convenient length of the corresponding expression) is given by

19 $$\begin{array}{rcl}\langle \langle \Psi (0)|\Psi (2\tau +\delta )\rangle \rangle  & = & +\frac{1}{\sqrt{2}}{e}^{-{\rm{i}}\tau {E}_{0}/h}{e}^{-{\rm{i}}\tau {E}_{0}/h}\langle 0|{\hat{u}}_{{\rm{vac}}}(\tau +\delta )|0\rangle \langle 0|{\hat{u}}_{{\rm{vac}}}(\tau +\delta )|0\rangle \\  &  & -\frac{1}{\sqrt{2}}{e}^{-{\rm{i}}\tau {E}_{1}/h}{e}^{-{\rm{i}}\tau {E}_{1}/h}\langle 1|{\hat{u}}_{{\rm{vac}}}(\tau +\delta )|1\rangle \langle 0|{\hat{u}}_{\alpha }(2\tau +\delta )|1\rangle \\  &  & +\frac{1}{\sqrt{2}}{e}^{-{\rm{i}}\tau {E}_{0}/h}{e}^{-{\rm{i}}\tau {E}_{1}/h}\langle 1|{\hat{u}}_{\alpha }(\tau +\delta )|0\rangle \langle 0|{\hat{u}^{\prime} }_{{\rm{vac}}}(2\tau +\delta )|1\rangle \\  &  & +\frac{1}{\sqrt{2}}{e}^{-{\rm{i}}\tau {E}_{0}/h}{e}^{-{\rm{i}}\tau {E}_{0}/h}\langle 0|{\hat{u}}_{{\rm{vac}}}(\tau +\delta )|0\rangle \langle 1|{\hat{u}}_{\alpha }(2\tau +\delta )|0\rangle \\  &  & -\frac{1}{\sqrt{2}}{e}^{-{\rm{i}}\tau {E}_{1}/h}{e}^{-{\rm{i}}\tau {E}_{1}/h}\langle 1|{\hat{u}}_{{\rm{vac}}}(\tau +\delta )|1\rangle \langle 1|{\hat{u}}_{{\rm{vac}}}(\tau +\delta )|1\rangle \end{array}$$

Note that the Eqs (10) and (17) reduce the problem of finding the probabilities to only the calculation of the matrix elements of the operators ${\hat{u}}_{{\rm{vac}}}$, ${\hat{u}}_{a}$, ${\hat{u}^{\prime} }_{vac}$ and ${\hat{u}^{\prime} }_{a}$. In this sense, some parity considerations are useful to realize which matrix elements are zero:

20a $$\langle m|{\hat{u}}_{{\rm{vac}}}|n\rangle ,\langle m|{\hat{u}^{\prime} }_{\alpha }|n\rangle =\{\begin{array}{ll}zero & :m-n\,{\rm{is}}\,{\rm{odd}}\\ non \mbox{-} zero & :m-n\,{\rm{is}}\,{\rm{even}}\end{array}$$

20b $$\langle m|{\hat{u}^{\prime} }_{{\rm{vac}}}|n\rangle ,\langle m|{\hat{u}}_{\alpha }|n\rangle =\{\begin{array}{ll}zero & :m-n\,{\rm{is}}\,{\rm{even}}\\ non \mbox{-} zero & :m-n\,{\rm{is}}\,{\rm{odd}}\end{array}$$

Accordingly, we obtain all nonvanishing matrix elements of the operators ${\hat{u}}_{{\rm{vac}}}(t)$, ${\hat{u}^{\prime} }_{{\rm{vac}}}(t)$, ${\hat{u}}_{a}(t)$ and ${\hat{u}^{\prime} }_{a}(t)$ as

21a $$\langle 0|{\hat{u}}_{{\rm{vac}}}(t)|0\rangle \simeq \exp [-\frac{{\rm{i}}}{\tilde{h}}\{t\delta {E}_{0}-|{f}_{10}{|}^{2}{F}_{+}(t)\}]$$

21b $$\langle 1|{\hat{u}}_{{\rm{vac}}}(t)|1\rangle \simeq \exp [-\frac{{\rm{i}}}{\tilde{h}}\{t\delta {E}_{1}-|{f}_{01}{|}^{2}{F}_{-}(t)\}]$$

21c $$\begin{array}{ccc}\langle 0|{\hat{u}^{\prime} }_{{\rm{vac}}}(t)|1\rangle  & \simeq  & \langle 0|{\hat{u}}_{\alpha }(t)|1\rangle \\  & = & {\langle 1|{\hat{u}}_{\alpha }(t)|0\rangle }^{\ast }\\  & = & \frac{2\pi {\rm{i}}}{\sqrt{2\tilde{h}}}{\bar{\gamma }}_{\alpha }{f}_{01}(\frac{1}{\pi })\frac{\sin (\omega +\Delta )t/2}{\omega +\Delta }{e}^{{\rm{i}}(\omega +\Delta )t/2}\end{array}$$

where ${F}_{\pm }(t)=-\,{\pi }^{(-1)}{\mathscr{P}}{\int }_{0}^{\infty }\,d\omega J(\omega )\frac{\sin (\omega \pm \Delta )t}{{(\omega \pm \Delta )}^{2}}$. Here the symbol ${\mathscr{P}}$ denotes that the integral preceded by it is a principal-value integral, $\Delta \,:\,=\frac{{E}_{1}-{E}_{0}}{\tilde{h}}$ is called the tunnel splitting of the ground-state energy and *J*(*ω*), namely the spectral function in the literature^38–40^, has the form $J(\omega )\,:\,=\frac{\pi }{2}{\{\bar{\gamma }(\omega )\}}^{2}D(\omega )$. The function *D*(*ω*) presents the frequency distribution of the environmental oscillators and *J*(*ω*) expresses the corresponding distribution weighted by the function ${\{\bar{\gamma }(\omega )\}}^{2}$ which describes the interaction strength. In our regime, *D*(*ω*) is defined as $D(\omega )\,:\,=\frac{1}{2\pi t}{\{\frac{\sin (\omega t/2)}{\omega /2}\}}^{2}$. Substituting all of the nonvanishing matrix elements into Eq. (19), we obtain the probability of finding the macrosystem in its initial state at time 2*τ* + *δ*, as

22 $$\begin{array}{rcl}|\langle \langle \Psi (0)|\Psi (2\tau +\delta )\rangle \rangle {|}^{2} & = & \frac{1}{2}(1-\Gamma \tau )+\frac{1}{2}(1-\Gamma \tau )\cos ({E}_{1}-{E}_{0})\tau /h\\  & = & \frac{1}{2}(1-\Gamma \tau )(1+\,\cos ({E}_{1}-{E}_{0})\tau /h)\end{array}$$

where *E*_0_ and *E*_1_ denote the energy of the levels |0〉 and |1〉, respectively, and Γ reflects the dissipation effect. That is,

23 $$\Gamma =\frac{2}{\tilde{h}}|{f}_{01}{|}^{2}J(\Delta ) \sim \frac{2}{\tilde{h}}|{f}_{01}{|}^{2}{\int }_{0}^{\infty }d\omega J(\omega )D(\omega -\Delta ;t)s$$

*J*(Δ) is a specified value of the spectral function. Here, it is worth pointing that Γ is the same quantity as called the effective decay rate in some previous studies^38,39^.

Furthermore, one can calculate the probability of finding the macrosystem in the initial state for other interaction steps, including the times *τ* + *δ*, 3*τ* + *δ* and 4*τ* + *δ*:

24a $$|\langle \langle \Psi (0)|\Psi (\tau +\delta )\rangle \rangle {|}^{2}=\frac{1}{2}(1-\frac{1}{2}\Gamma \tau )(1+\,\cos ({E}_{1}-{E}_{0})\tau /h)$$

24b $$|\langle \langle \Psi (0)|\Psi (3\tau +\delta )\rangle \rangle {|}^{2}=\frac{1}{2}(1-\frac{3}{2}\Gamma \tau )(1+\,\cos \,3({E}_{1}-{E}_{0})\tau /h)$$

24c $$|\langle \langle \Psi (0)|\Psi (4\tau +\delta )\rangle \rangle {|}^{2}=\frac{1}{2}(1-2\Gamma \tau )(1+\,\cos \,4({E}_{1}-{E}_{0})\tau /h)$$

here, we avoid to mention the details of evaluating the expression 〈Ψ(0)|Ψ(4*τ* + *δ*)〉 due to the very long calculations, but you can see it in detail in Appendix B. As a result, a prediction for the *N*th interaction step arises from comparing the results for the survival probability at different times, i.e., the Eqs (22), (24a) and (24b). Thus we arrive at

25 $$|\langle \langle \Psi (0)|\Psi (N\tau +\delta )\rangle \rangle {|}^{2}=\frac{1}{2}{e}^{-\frac{N}{2}\Gamma \tau }(1+\,\cos \,N({E}_{1}-{E}_{0})\tau /h)$$

We define the total evolution time as *T*:= *Nτ* and rewrite the Eq. (25) as

26 $${P}_{tot}(T)=|\langle \langle \Psi (0)|\Psi (T)\rangle \rangle {|}^{2}=\frac{1}{2}{e}^{-\frac{1}{2}\Gamma T}(1+\,\cos ({E}_{1}-{E}_{0})T/h)$$

Defining ${\tau }_{Q}:=\frac{1}{\Delta }$ and expanding the exponential and cosine functions up to second order, we arrive at

27 $${P}_{tot}(T)\simeq 1-\frac{1}{2}\Gamma T-\frac{1}{4}{(\frac{T}{{\tau }_{Q}})}^{2}$$

where higher order terms are neglected. Recall that Δ is the tunneling amplitude, so *τ*_*Q*_ is the corresponding characteristic time called the quantum tunneling time. Note that *P*_*tot*_(*T*) is the probability of finding the total system including the macrosystem-environment couple in the initial state. However, the quantity of interest, usually, is not *P*_*tot*_(*T*) but is the probability of remaining the macrosystem in its initial state, which we denote as *P*_*S*_(*T*):

28 $${P}_{S}(T)=|\langle \langle \psi ,{\rm{vac}}|\Psi (T)\rangle \rangle {|}^{2}+\sum _{\alpha }\,|\langle \langle \psi ,\alpha |\Psi (T)\rangle \rangle {|}^{2}$$

Now, it is obvious that

29 $$1\ge {P}_{S}(T)\ge {P}_{tot}(T)$$

Combining the above inequality with Eq. (27), we finally arrive at the following inequality

30 $$0\le 1-{P}_{S}(T)\le \frac{1}{2}\Gamma T+\frac{1}{4}{(\frac{T}{{\tau }_{Q}})}^{2}$$

Equation (30) indicates that as *τ* → 0, the term on right-hand side vanishes, which implies that *P*_*S*_(*T*) → 1. Thus the evolution of the macrosystem becomes frozen in this limit, so it may be interpreted as QZE. If the macrosystem interacts with the environment frequently enough and the interaction time intervals are also small enough, Eq. (30) ensures that the macrosystem remains in the initial state as before.

## The Effect of Quasi-Classical Situation on Zeno Behavior

In this section, we first introduce the dimensionless parameter $\tilde{h}$ which quantifies the extent to which a system is expected to behave as a macroscopic one. Thence, we investigate the behavior of the decay function Γ(*τ*) as the system adopts different degrees of macroscopicity. We follow the approach which introduced by Takagi in 1997^40^.

Without loss of generality one can suppose that for a macroscopic two-level system, the potential has the characteristic length *R*_0_ with the unit of length and the characteristic energy *U*_0_ with the unit of energy. Moreover, the corresponding characteristic time *τ*_0_ may be introduced as the time required for a particle of mass *M* to pass the distance *R*_0_ at a constant speed with the kinetic energy of the order of *U*_0_. So, we have

31 $${\tau }_{0}\,:\,=\frac{{R}_{0}}{{({U}_{0}/M)}^{1/2}}$$

It is possible to determine *τ*_0_ by the height and the width of the energy barrier, so it is usually called the tunneling time. Here, we consider *τ*_0_ as the unit of time. Now, we introduce the parameter $\tilde{h}$, which instead of Planck’s constant appears in a particular dimensionless form of the Schrödinger equation resulting from our choice of units:

32 $$\tilde{h}\,:\,=\frac{\hslash }{{U}_{0}{\tau }_{0}}=\frac{\hslash }{{P}_{0}{R}_{0}}={\{\frac{{\hslash }^{2}/M{{R}_{0}}^{2}}{{U}_{0}}\}}^{1/2}$$

where *P*_0_ is the unit of momentum defined as *P*_0_:= (*MU*_0_)^1/2^. The magnitude of $\tilde{h}$ is related to *U*_0_*τ*_0_(= *P*_0_*R*_0_) and determines how much a system behaves as a macroscopic quantum-mechanical one. In this sense, the condition in which $\tilde{h}\ll 1$ is called the quasi-classical situation. One with a purely quantum-mechanical approach prefers to work with $\tilde{h}=1$, but one with a classical approach tracks $\tilde{h}$ to indicate where the quantum mechanical behavior starts to appear. If $\tilde{h}$ is too small, it is impossible to detect quantum effects. For a given macroscopic system we consider its typical value is in the range $0.01\le \tilde{h}\le 0.1$.

Now let us examine how the change in the macroscopicity affects the decay function Γ(*t*), thus consequently modifies the Zeno and anti-Zeno behaviors. To do this, we employ an explicit expression for *J* as $J(\omega )=\eta \omega {(\frac{\omega }{{\omega }_{c}})}^{s-1}\exp (\,-\,\omega /{\omega }_{c})$, where *s* characterizes the Ohmicity of the environment, *ω*_*c*_ is the cutoff frequency, and *η* is a positive constant which we consider to quantify the macrosystem-environment interaction. Environments are classified based on the value of *s* as the following^40^

$$0 < s < 1\,:{\rm{sub}} \mbox{-} {\rm{Ohmic}}\,{\rm{environment}},$$

$$s={\rm{1}}\,:{\rm{Ohmic}}\,{\rm{environment}},$$

$$s > {\rm{1}}\,:{\rm{super}} \mbox{-} {\rm{Ohmic}}\,{\rm{environment}}.$$

**Figure 2:**
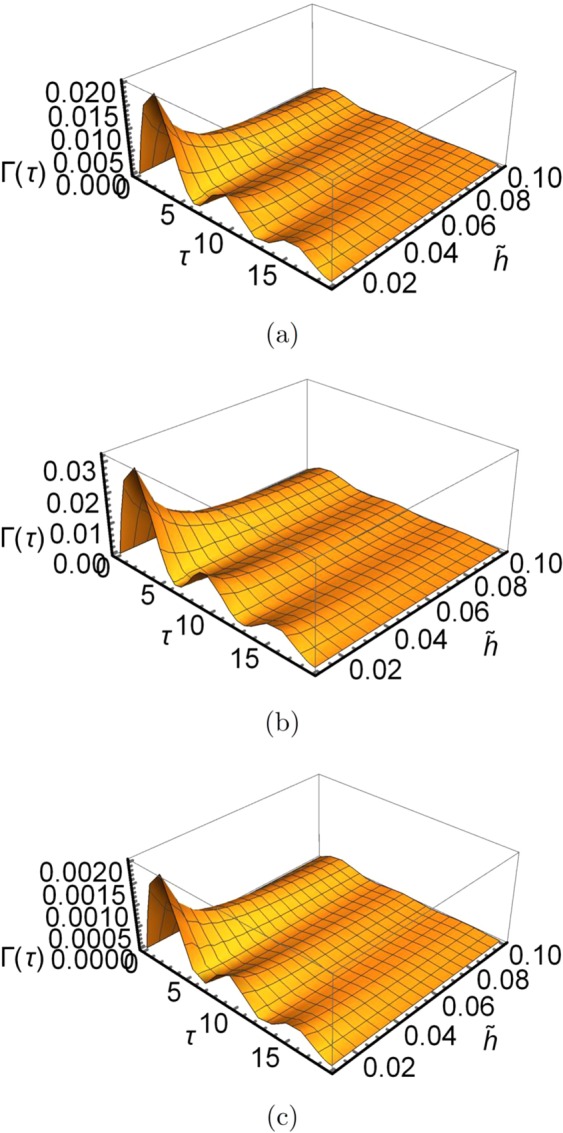
Variation of the decay rate for a given macroscopic quantum system, (**a**) plot of Γ(*τ*) as a function of *τ* and $\tilde{h}$ with Δ = 1, *η* = 0.001, *ω*_*c*_ = 10 for an Ohmic environment (s = 1), (**b**) Same as (**a**) but now we have a sub-Ohmic environment with *s* = 0.8, (**c**) Same as (**a**) but now we have a super-Ohmic situation with *s* = 2.

The function Γ(*τ*) can be represented as $\Gamma (\tau )=\frac{1}{\pi \tilde{h}}|{f}_{01}{|}^{2}\eta {\int }_{0}^{\infty }d\omega {(\frac{\omega }{{\omega }_{c}})}^{s-1}\frac{\omega }{t}\exp (\,-\,\omega /{\omega }_{c}){\{\frac{\sin (\omega t/2)}{\omega /2}\}}^{2}$ by substituting the the functions *J*(*ω*) and *D*(*ω*) into Eq. 23. In Fig. 2a to c, we have illustrated the variation of Γ(*τ*) as the function of the interaction interval *τ* and the macroscopicity $\tilde{h}$, where $\tilde{h}$ changes from 0.01 to 0.1. The Ohmicity of the environment takes different values in each case, *s* = 1, *s* = 0.8 and *s* = 2, for Fig. 2a–c, respectively. According to Fig. 2, with the chosen values of the system-environment parameters, we see the transitions between Zeno and anti-Zeno regime occur as *τ* increases. When the graph of Γ(*τ*) possesses a positive slope versus increasing *τ*, we stand in the Zeno regime. On the opposite side, when the gradient becomes minus, we lie in the anti-Zeno situation. According to this figure, there are multiple Zeno and anti-Zeno regions, which may be interpreted as’multiple extrema’ behavior of Γ(*τ*)^38,39^. Furthermore, we see that for larger $\tilde{h}$, the decay function Γ(*τ*) takes the smaller values. This situation is entirely consistent with what we expect for a system with more quantum mechanical behavior. For a system with a more classical trait, the decay rate takes larger values. Thus, the system-environment interaction decays faster than a quantum system.

**Figure 3:**
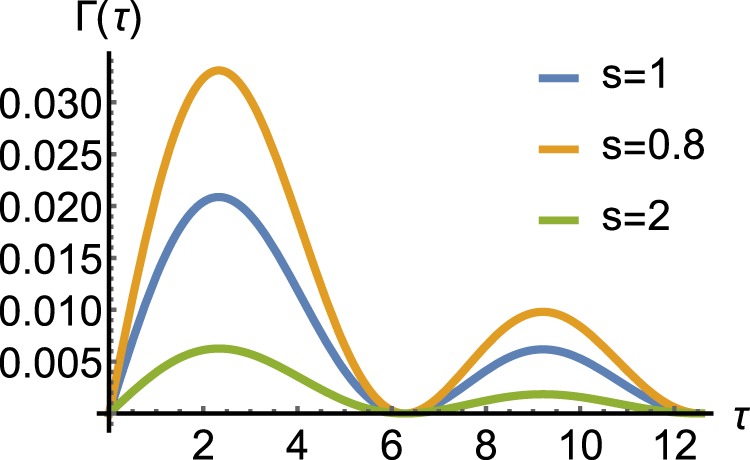
Variation of the decay rate for a given macroscopic quantum system with $\tilde{h}=0.01$. We choose the parameters Δ = 1, *η* = 0.001, *ω*_*c*_ = 10. The Ohmicity of the environment is *s* = 1, *s* = 0.8 and *s* = 2 for the *blue*, *orange* and *green* curves, respectively.

**Figure 4:**
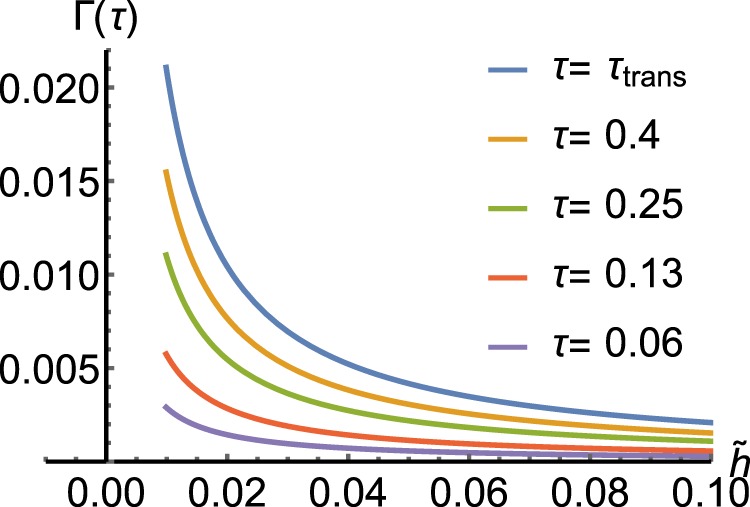
Variation of the decay rate for a given macroscopic quantum system vs $\tilde{h}$ at different times. Here, we have set Δ = 1, *η* = 0.001, *ω*_*c*_ = 10 and *s* = 1.

Figure (3) shows the variation of the Γ(*τ*) for a system with $\tilde{h}=0.01$ and different values of Ohmicity. We see the change in the strength of the system-environment interaction alters the decay rate, quantitatively. But the time of the Zeno to anti-Zeno transition remains unchanged, approximately. In Fig. (4) one can see the variation of Γ(*τ*) vs $\tilde{h}$ where $\tilde{h}$ changes continuously from 0.01 to 0.1, for typical environmental parameters, at different times. Accordingly, Γ(*τ*) has the largest value and the sharpest variation when the interaction time interval is equal to the QZE to QAZE transition time. Also, we can see an explicit distinction between the behavior of a quantum and classical object in the case of the exhibition of QZE.

## Conclusion

We have worked out a new approach to study the Zeno behavior for a two-level macroscopic quantum system under the successive and step-by-step interactions with a harmonic environment. We have used perturbation theory, due to the weak interactions between the system and the harmonic environment. We have derived a new expression for the probability of remaining the macrosystem in its initial state, after the *N*th interaction step, which is different from the well-known survival probability *S* = *e*^−Γ(*τ*)*Nτ*^. Multiple transitions between Zeno and anti-Zeno behaviors are detectable in our formalism. We have shown that along with some environmental parameters like the Ohmicity, the macroscopic trait of a system has a notable effect on the decay function Γ(*τ*).

## Supplementary information

Appendices
