# Supplementary material for: A new approach to study the Zeno effect for a macroscopic quantum system under frequent interactions with a harmonic environment
Source: Sci Rep. 2019 Oct 24;9:15265. doi: 10.1038/s41598-019-51729-1 (PMC6813307; doi:10.1038/s41598-019-51729-1)
Supplement: Supplementary file 1 — Appendices [file 41598_2019_51729_MOESM1_ESM.pdf]

# A new approach to study the Zeno effect for a macroscopic quantum system under frequent interactions with a harmonic environment

Fatemeh Ghasemi and Afshin Shafiee

## Appendix A: Additional Relations and Variables

The operators $\hat{u}_{\mathrm{vac}}(t)$, $\hat{u}_\alpha(t)$, $\hat{u}'_{\mathrm{vac}}(t)$ and $\hat{u}'_\alpha(t)$ are defined as

$$\begin{aligned}\hat{u}_{\mathrm{vac}}(t) &:= 1 - \frac{\mathrm{i}}{\tilde{h}}\int_0^t \mathrm{d}t_1 \delta\hat{V}(t_1) \\ &- \frac{1}{2\tilde{h}}\sum_\alpha \int_0^t \mathrm{d}t_2 \int_0^{t_2} \mathrm{d}t_1 \hat{f}_\alpha(t_2){\omega_\alpha}^3 \mathrm{e}^{-\mathrm{i}(t_2-t_1)\omega_\alpha}\hat{f}_\alpha(t_1) \end{aligned} \tag{A1}$$

$$\hat{u}_\alpha(t) := \frac{\mathrm{i}}{\sqrt{2\tilde{h}}}\int_0^t \mathrm{d}t_1 \hat{f}_\alpha(t_1){\omega_\alpha}^{3/2}\mathrm{e}^{-\mathrm{i}\omega_\alpha t_1} \tag{A2}$$

$$\begin{aligned}\hat{u}'_{\mathrm{vac}} &:= +\sqrt{\frac{\tilde{h}}{2}}\left(\frac{\mathrm{i}}{\tilde{h}}\right)\int_0^t \mathrm{d}t_1 {\omega_\alpha}^{3/2}\hat{f}_\alpha(t_1)\mathrm{e}^{-\mathrm{i}\omega_\alpha t_1} \\ &- \frac{1}{2\tilde{h}}\int_0^t \mathrm{d}t_2 \int_0^{t_2} \mathrm{d}t_1 \hat{f}_\alpha(t_2)\mathrm{e}^{-\mathrm{i}(t_2-t_1)\omega_\alpha}{\omega_\alpha}^{7/2}\hat{f}_\alpha(t_1) \end{aligned} \tag{A3}$$

$$\begin{aligned}\hat{u}'_\alpha(t) &:= 1 - \frac{\mathrm{i}}{2\tilde{h}}\sum_\alpha \int_0^t \mathrm{d}t_1 {\omega_\alpha}^2 \hat{f}^2_\alpha(t_1) \\ &- \frac{1}{2\tilde{h}}\sum_\alpha \int_0^t \mathrm{d}t_2 \int_0^{t_2} \mathrm{d}t_1 \hat{f}_\alpha(t_2)\mathrm{e}^{-\mathrm{i}(t_2-t_1)\omega_\alpha}{\omega_\alpha}^3 \hat{f}_\alpha(t_1) \end{aligned} \tag{A4}$$

## Appendix B: The probability amplitiude at $(4\tau + \delta)$

We evaluate the expression $\langle\langle\Psi(0)|\Psi(4\tau+\delta)\rangle\rangle$ as the following:

$$
\begin{aligned}
\langle\langle\Psi(0)|\Psi(4\tau+\delta)\rangle\rangle =& + \frac{1}{2}e^{-\mathrm{i}4\tau E_0/\tilde{h}}\langle 0|\hat{u}_{\mathrm{vac}}|0\rangle^4 \\
& - \frac{1}{2}e^{-\mathrm{i}4\tau E_0/\tilde{h}}\langle 0|\hat{u}_{\mathrm{vac}}|0\rangle^3\langle 1|\hat{u}'_{\alpha}|0\rangle \\
& - \frac{1}{2}e^{-\mathrm{i}2\tau E_0/\tilde{h}}e^{-\mathrm{i}2\tau E_1/\tilde{h}}\langle 0|\hat{u}_{\alpha}|1\rangle\langle 0|\hat{u}'_{\alpha}|0\rangle\langle 0|\hat{u}'_{\mathrm{vac}}|1\rangle\langle 0|\hat{u}'_{\alpha}|1\rangle \\
& + \frac{1}{2}e^{-\mathrm{i}2\tau E_0/\tilde{h}}e^{-\mathrm{i}2\tau E_1/\tilde{h}}\langle 0|\hat{u}_{\alpha}|1\rangle\langle 0|\hat{u}'_{\alpha}|0\rangle\langle 1|\hat{u}'_{\mathrm{vac}}|0\rangle\langle 1|\hat{u}_{\mathrm{vac}}|1\rangle \\
& + \frac{1}{2}e^{-\mathrm{i}3\tau E_0/\tilde{h}}e^{-\mathrm{i}\tau E_1/\tilde{h}}\langle 1|\hat{u}_{\alpha}|0\rangle\langle 0|\hat{u}'_{\mathrm{vac}}|1\rangle\langle 0|\hat{u}_{\mathrm{vac}}|0\rangle^2 \\
& - \frac{1}{2}e^{-\mathrm{i}3\tau E_0/\tilde{h}}e^{-\mathrm{i}\tau E_1/\tilde{h}}\langle 1|\hat{u}_{\alpha}|0\rangle\langle 0|\hat{u}'_{\mathrm{vac}}|1\rangle\langle 0|\hat{u}_{\mathrm{vac}}|0\rangle\langle 1|\hat{u}_{\alpha}|0\rangle \\
& - \frac{1}{2}e^{-\mathrm{i}2\tau E_0/\tilde{h}}e^{-\mathrm{i}2\tau E_1/\tilde{h}}\langle 1|\hat{u}_{\mathrm{vac}}|1\rangle\langle 0|\hat{u}_{\alpha}|1\rangle\langle 0|\hat{u}_{\mathrm{vac}}|0\rangle^2 \\
& + \frac{1}{2}e^{-\mathrm{i}2\tau E_0/\tilde{h}}e^{-\mathrm{i}2\tau E_1/\tilde{h}}\langle 1|\hat{u}_{\mathrm{vac}}|1\rangle\langle 0|\hat{u}_{\alpha}|1\rangle\langle 0|\hat{u}_{\mathrm{vac}}|0\rangle\langle 1|\hat{u}_{\alpha}|0\rangle \\
& + \frac{1}{2}e^{-\mathrm{i}3\tau E_0/\tilde{h}}e^{-\mathrm{i}\tau E_1/\tilde{h}}\langle 0|\hat{u}_{\mathrm{vac}}|0\rangle\langle 1|\hat{u}_{\alpha}|0\rangle\langle 0|\hat{u}'_{\mathrm{vac}}|1\rangle\langle 1|\hat{u}_{\mathrm{vac}}|1\rangle \\
& - \frac{1}{2}e^{-\mathrm{i}3\tau E_0/\tilde{h}}e^{-\mathrm{i}\tau E_1/\tilde{h}}\langle 0|\hat{u}_{\mathrm{vac}}|0\rangle\langle 1|\hat{u}_{\alpha}|0\rangle\langle 0|\hat{u}'_{\mathrm{vac}}|1\rangle\langle 1|\hat{u}_{\alpha}|0\rangle \\
& - \frac{1}{2}e^{-\mathrm{i}\tau E_0/\tilde{h}}e^{-\mathrm{i}3\tau E_1/\tilde{h}}\langle 0|\hat{u}_{\alpha}|1\rangle\langle 1|\hat{u}'_{\mathrm{vac}}|0\rangle\langle 1|\hat{u}_{\mathrm{vac}}|1\rangle\langle 0|\hat{u}_{\alpha}|1\rangle \\
& + \frac{1}{2}e^{-\mathrm{i}\tau E_0/\tilde{h}}e^{-\mathrm{i}3\tau E_1/\tilde{h}}\langle 0|\hat{u}_{\alpha}|1\rangle\langle 1|\hat{u}'_{\mathrm{vac}}|0\rangle\langle 1|\hat{u}_{\mathrm{vac}}|1\rangle^2 \\
& + \frac{1}{2}e^{-\mathrm{i}2\tau E_0/\tilde{h}}e^{-\mathrm{i}2\tau E_1/\tilde{h}}\langle 1|\hat{u}_{\alpha}|0\rangle\langle 1|\hat{u}'_{\alpha}|1\rangle\langle 0|\hat{u}'_{\mathrm{vac}}|1\rangle\langle 0|\hat{u}_{\mathrm{vac}}|0\rangle \\
& - \frac{1}{2}e^{-\mathrm{i}2\tau E_0/\tilde{h}}e^{-\mathrm{i}2\tau E_1/\tilde{h}}\langle 1|\hat{u}_{\alpha}|0\rangle\langle 1|\hat{u}'_{\alpha}|1\rangle\langle 0|\hat{u}'_{\mathrm{vac}}|1\rangle\langle 1|\hat{u}_{\alpha}|0\rangle \\
& - \frac{1}{2}e^{-\mathrm{i}4\tau E_1/\tilde{h}}\langle 1|\hat{u}_{\mathrm{vac}}|1\rangle^3\langle 0|\hat{u}_{\alpha}|1\rangle \\
& + \frac{1}{2}e^{-\mathrm{i}4\tau E_1/\tilde{h}}\langle 1|\hat{u}_{\mathrm{vac}}|1\rangle^4
\end{aligned}
\tag{B1}
$$
